# Family physicians' perceptions of academic detailing: a quantitative and qualitative study

**DOI:** 10.1186/1472-6920-7-36

**Published:** 2007-10-12

**Authors:** Michael Allen, Suzanne Ferrier, Nicolette O'Connor, Isobel Fleming

**Affiliations:** 1 Continuing Medical Education, Dalhousie University, Clinical Research Centre, 5849 University Avenue, Halifax, Nova Scotia, B3H 4H7, Canada; 2 Department of Political Science, Carleton University, B640 Loeb Building, 1125 Colonel By Drive, Ottawa, Ontario, K1S 5B6, Canada

## Abstract

**Background:**

The efficacy of academic detailing in changing physicians' knowledge and practice has been the subject of many primary research publications and systematic reviews. However, there is little written about the features of academic detailing that physicians find valuable or that affect their use of it. The goal of our project was to explore family physicians' (FPs) perceptions of academic detailing and the factors that affect their use of it.

**Methods:**

We used 2 methods to collect data, a questionnaire and semi-structured telephone interviews. We mailed questionnaires to all FPs in the Dalhousie Office of Continuing Medical Education database and analyzed responses of non-users and users of academic detailing. After a preliminary analysis of questionnaire data, we conducted semi-structured interviews with 7 FPs who did not use academic detailing and 17 who did use it.

**Results:**

Overall response rate to the questionnaire was 33% (289/869). Response rate of non-users of academic detailing was 15% (60/393), of users was 48% (229/476). The 3 factors that most encouraged use of academic detailing were the topics selected, the evidence-based approach adopted, and the handout material. The 3 factors that most discouraged the use of academic detailing were spending office time doing CME, scheduling time to see the academic detailer, and having CME provided by a non-physician. Users of academic detailing rated it as being more valuable than other forms of CME. Generally, interview data confirmed questionnaire data with the exception that interview informants did not view having CME provided by a non-physician as a barrier. Interview informants mentioned that the evidence-based approach adopted by academic detailing had led them to more critically evaluate information from other CME programs, pharmaceutical representatives, and journal articles, but not advice from specialists.

**Conclusion:**

Users of academic detailing highly value its educational value and tend to view information from other sources more critically because of its evidence-based approach. Non-users are unlikely to adopt academic detailing despite its high educational value because they find using office time for CME too much of a barrier. To reach these physicians with academic detailing messages, we will have to find other CME formats.

## Background

Academic detailing or educational outreach is a form of continuing medical education (CME) in which a trained health professional such as a physician or pharmacist visits physicians in their offices to provide evidence-based information. The efficacy of academic detailing in changing physicians' knowledge and practice has been the subject of many primary research publications and systematic reviews. The most recent review found that academic detailing in conjunction with other educational interventions led to a median improvement in physician performance of approximately 6.0%[1]. Several studies have found that physicians rate the educational value of academic detailing highly [2-4].

Despite the number of studies about the efficacy of academic detailing, few have addressed the features of academic detailing that physicians find valuable or that affect their use of it. Habraken et al. found that Belgian physicians highly rated academic detailing visits and approximately 90% of those who used academic detailing wished to use it again[4]. However, they also identified some barriers to participation: the information was not new or could be obtained in other ways, the information was politically coloured and designed to cut expenses, and the educational visits were time-consuming[5]. The goal of our project was to explore family physicians' (FPs) perceptions of academic detailing and the factors that affect their use of it.

The Office of CME at Dalhousie University has had an Academic Detailing Service since 2001. Funded by the provincial Department of Health, the Service is available to all FPs in Nova Scotia (approximately 850 are in practices for which academic detailing is relevant, e.g., in active family practice, not in solely administrative or emergency medicine roles). Three academic detailers, 2 pharmacists and a nurse, present 1 or 2 evidence-based topics per year. Topics are selected by surveying FPs, by scanning the literature for areas of interest, and to complement other provincial health initiatives. We research the evidence for each topic with the help of a drug evaluation pharmacist. A specialist physician and advisory board of 4 FPs ensure that the evidence-based information is clinically relevant. If a physician poses a question that the academic detailer cannot answer during the visit, the academic detailing team finds an answer and faxes the response.

During our detailing session, we present data from clinical trials in absolute as well as relative terms and include event rates of active and placebo groups, absolute risk reduction, and numbers needed to treat with 95% confidence intervals. We believe this approach presents a more accurate estimate of treatment effect than presenting data in only relative terms and there is evidence that the way data is presented affects prescribing decisions [6-8]. During our visits and in our handout material, we explain these terms to physicians. Most visits last about 25 minutes, are with individual physicians, take place during regular working hours, and provide 1 MAINPRO M-1 credit of the College of Family Physicians of Canada[9].

Handout material left with physicians consists of a booklet of up to 40 pages that provides details of clinical trial evidence. A few pages of summary statements in the front of the booklet summarize the key points of the evidence. We also leave double-sided laminated sheet that contains essential points for ready reference. Examples of handout material are at http://cme.medicine.dal.ca/ADS.htm.

For each topic, about 360 FPs use the service. By 2004, our records showed that approximately 43% of FPs had never used academic detailing, 14% had used it once, and 43% had used it more than once. We wished to determine the factors that encourage and discourage FPs from using academic detailing. Our research questions were:

1. What features of academic detailing

• encourage physician participation?

• discourage physician participation?

• do physicians find valuable?

2. How can academic detailing be improved to better meet the CME needs of physicians?

3. What is the value of academic detailing compared to other forms of CME?

## Methods

This was a mixed-methods study[10] using 2 methods to collect data, a questionnaire and semi-structured telephone interviews. For both methods, we divided our study population into 3 groups of FPs based on their participation: those who had never used academic detailing (used never group), those who had used academic detailing once (used once group), and those who had used academic detailing more than once (used > once group). Nova Scotia FPs in practice, regardless of group, were sent invitations to participate in previous academic detailing sessions. The Research Ethics Board of Dalhousie University approved the project.

### Questionnaire

Two of the authors involved in the Dalhousie Academic Detailing Service (MA, IF) and two colleagues (see acknowledgements) who have a strong background in educational theory and questionnaire design developed the questionnaire. There is little published information on the factors that encourage and discourage physicians from using academic detailing. Therefore we developed questions to address factors that our experience and informal discussion with physicians indicated may be important. Four physicians from each group tested the questionnaire for face validity.

An introductory letter described features of the Academic Detailing Service. The three-page questionnaire collected demographic and practice information and asked respondents to rate on a five-point Likert scale how much various factors encouraged or discouraged use of academic detailing, and how likely they were to use academic detailing in the future. Open-ended questions asked for suggestions to make academic detailing better meet respondents' learning needs and for general comments. The questionnaires for the 3 groups were identical except for 1 question. The used never group were asked if they had heard about the academic detailing service before receiving the questionnaire while the other 2 groups were asked to rate the value of academic detailing compared to other forms of CME.

In September 2004 we mailed the questionnaire to all FPs in the Dalhousie CME database whom we considered eligible to participate in academic detailing (N = 869). We offered a chance to win two $50 vouchers as an incentive and re-mailed the questionnaire to non-responders 3 weeks after the initial mailing. The questionnaire also asked respondents to indicate if they were interested in being interviewed.

Questionnaire data were analyzed using SPSS version 10.1. We calculated descriptive statistics for data collected in Likert scales, and frequency distributions for non-continuous data. Means and frequency distributions of the study groups were compared using inferential statistics (i.e., analysis of variance and chi-square tests, respectively). We set an alpha level of 0.004 with a Bonferroni correction to adjust for the number of encouraging and discouraging factors being compared.

### Telephone interviews

We planned 10 interviews with each group of physicians and developed the interview questions after a preliminary analysis of the questionnaire data to determine themes for exploration (see Additional file 1 for questions). We randomly selected physicians from questionnaire respondents who expressed interest in being interviewed. We tape recorded and transcribed interviews and mailed the transcriptions to subjects to verify accuracy.

Using a thematic content analysis, the interview data were coded, or broken down, into manageable categories and then examined for the frequency of occurrences of each code. Interview transcripts were analyzed independently by researchers (MA and NO), and reviewed by a third researcher (SF). The coding of the researchers were compared, and in the case of discrepancies, the researchers reviewed and discussed the text until agreement was reached. We used QSR NUD*IST 6 for data management.

## Results

We received only 24 questionnaire responses from the used once group, a response rate of 25%. These responses were not significantly different from the used > once group so we combined the 2 for data analysis. Similarly, for interviews we were able to schedule only 5 interviews with the used once group. Their responses were not different from the used > once group and so we combined them for analysis. Therefore, we ended up with 2 groups, those who did use and did not use the academic detailing service (users and non-users respectively).

### Questionnaire quantitative results

Table 1 shows the response rate of the 2 groups. The overall response rate was 33% though it varied widely between groups. Table 2 shows demographic and practice data. Forty-two percent of respondents were female and approximately 60% were members of the College of Family Physicians of Canada. Significantly more non-user respondents came from communities with populations larger than 50,000 and significantly more user respondents came from communities smaller than 5,000. The questionnaire respondents were similar in terms of gender and year of graduation to other physicians who received the questionnaire.

**Table 1:** Response rate to questionnaire

**Study group**	**Mailed**	**Returned**	**Percent returned**
**Non-users**	393	60	15
**Users**	476	229	48
**Total**	869	289	33

**Table 2:** Demographic and practice information of questionnaire respondents

	**Non-users**		**Users**	
	**N**	**%**	**N**	**%**

Female	25	42	97	42
Member of CFPC*	34	57	140	61
Size of community+				
<5000	6	10	59	27
5,000 to 50,000	21	36	82	37
>50,000	32	54	79	36
				
Number patients seen per week N (SD)	121 (72)		127 (56)	
Practice hours per week				
1 to 20	15	25	34	15
21 to 35	12	20	46	21
36 to 50	21	36	86	39
>50	10	17	53	24
				
Year of graduation				
1969 or earlier	8	14	19	8
1970 to 1979	12	20	72	32
1980 to 1989	23	39	78	34
1990 to 1999	14	24	48	21
2000 to 2004	2	3	6	3

*College of Family Physicians of Canada

+ Statistically significant difference at p < 0.01

Table 3 shows responses to questions asking physicians to rate the factors that encourage and discourage their use of academic detailing. Ratings of users were significantly higher than non-users at the p < 0.004 level for all factors except adopting an evidence-based approach, having the detailer follow up by answering questions, and obtaining CME credits.

**Table 3:** Ratings on questionnaire to factors that encourage and discourage family physicians' use of academic detailing (using a 5-point Likert scale where 1 = strongly discourage and 5 = strongly encourage)

	**Non-users**	**Users**
**Encouraging/Discouraging Factor**	**Mean Rating (SD)**	**Mean Rating (SD)**

Relevance to practice of topic being presented	3.63 (1.1)	4.45 (0.6)*
Evidence-based approach	4.00 (1.1)	4.38 (0.8)
Usefulness of handout	3.27 (1.3)	4.30 (0.8)*
Effectiveness of academic detailing as a way of learning	3.21 (1.4)	4.18 (0.9)*
Awareness that topic was being presented	3.19 (1.0)	4.11 (0.8)*
Follow up by finding answers to questions	3.86 (1.0)	4.02 (0.9)
Clinical knowledge of topic being presented	3.40 (1.0)	3.84 (0.8)*
Obtaining CME credits	3.89 (0.9)	3.67 (1.0)
Scheduling time to see the academic detailer	2.52 (1.3)	3.45 (1.0)*
Having access to CME in other ways	3.02 (1.2)	3.36 (0.9)*
Spending office time doing CME	2.11 (1.1)	3.33 (1.0)*
Having CME provided by a non-physician	2.55 (1.0)	3.21 (0.8)*

The body of the question was: Many factors may determine whether physicians see an academic detailer for CME. Please rate how much the following aspects of the Dalhousie Academic Detailing Service discourage or encourage you from seeing an academic detailer.

* Statistically significant at p < .004

For non-users, the factors that most discouraged them from using academic detailing were spending office time doing CME, scheduling office time to see an academic detailer, and having CME provided by a non-physician. The mean rating of each of these domains was less than 3.0 indicating that these factors may actually be a deterrent to participating in academic detailing. For the users, the factors with the lowest ratings were having CME provided by a non-physician, spending office time doing CME, and having access to CME in other ways. The mean rating of each of these domains was approximately 3.0 indicating that these factors were neither encouraging nor discouraging.

For users, the 3 factors that most encouraged the use of academic detailing were the relevance of the topic, the evidence-based approach adopted in academic detailing, and the usefulness of the handout material. For non-users, the most encouraging factors were the evidence-based approach, obtaining CME credits, and having the detailer follow up by answering questions. Mean ratings for most encouraging factors were ≥ 4.3 for the users and 3.9 to 4.0 for the non-users.

**Figure 1:**
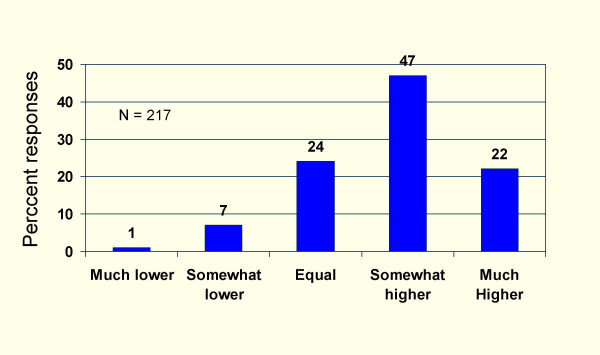
Users' rating of academic detailing compared to other forms of CME.

**Figure 2:**
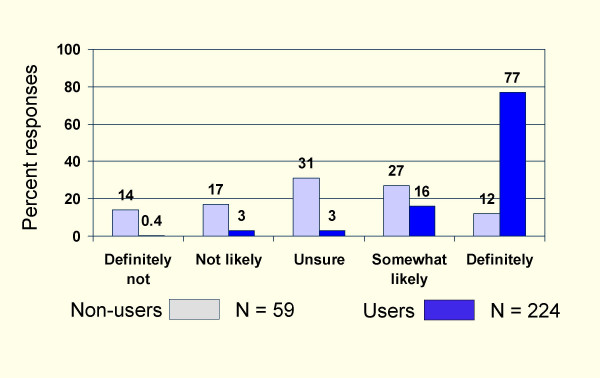
Percent of questionnaire respondents likely to use the Academic Detailing Service in the future.

Figure 1 shows that 68% of user respondents rated academic detailing as being of higher or much higher value than other forms of CME. Figure 2 shows that 93% of users were somewhat likely to use or would definitely use academic detailing in the future compared to 39% of non-users (p < 0.0001).

### Interviews and responses to open ended questions on questionnaire

Forty-one non-users and 88 users made comments to open-ended questions on the questionnaire. We were able to recruit 7 non-users and 17 users for interviews. In reporting qualitative data, we will concentrate on some factors that most discouraged and encouraged physicians from using academic detailing.

This section reports qualitative data from both the interviews and questionnaires. If the source is not specified, it is from the interview participants.

### Scheduling and spending office time for CME

All non-users except 1 indicated that scheduling was a concern. One non-user mentioned he had nothing against academic detailing, but just did not have the spare time. In another practice with 2 physicians, 1 non-user said it may be possible to see an academic detailer at lunchtime but he might be called away for an emergency. In reply to a question about what might make them use academic detailing, 2 non-user interview participants had no suggestions because there simply was not enough time. In contrast, only 1 user found scheduling a time a barrier. Other users found no difficulty.

"It's better than spending office time seeing commercial detailers or spending evenings so it's not a problem. It's not an onerous imposition."

One user found it less than ideal but still a practical approach to learning considering other demands.

"It is not ideal because it means that we tend to be in a rush, and are often coming from seeing a patient, and maybe haven't had lunch. We tend to cram it into a busy day, which isn't really the best way to learn. But there isn't really much other time. So I don't think it is ideal but I think it is a reasonably practical approach to how much time people have."

On the questionnaire, 6 respondents from the users made comments about difficulty with scheduling while 2 reported no difficulty. Questionnaire and interview data indicated that the preferred times for seeing the academic detailer were in the morning before seeing patients, at noon, and at the end of the day.

### CME provided by a non-physician

Only 1 non-user considered having non-physicians as detailers a concern in interviews while 3 non-users made comments on the questionnaire.

"I find it offensive having a non-MD presenting this information. Their lack of training in physiology and pathology of diseases makes their input useless."

Two users expressed some concern that non-physicians could not answer their questions at the time of the visit and 1 other user was unsure if it would be a wise use of physicians' time for them to be detailing. Comments from other interview informants were generally favorable about having non-physicians present CME.

"Drug company reps may have an undergrad science degree but no medical background. I'm quite happy to get it from a pharmacist. I work with nurses and have no problem."

"As long as they identify their area of expertise. And so far the detailing that we've gotten, I feel confident in the presenters. And I am actually quite impressed that they seem to have quite a good knowledge of the topics."

### Topic selection

In interviews, 3 non-users mentioned that selection of relevant topics might lead to them using academic detailing. One suggested that we choose topics that pharmaceutical companies are concentrating on while on the questionnaire 1 respondent suggested we cover topics that were not usually presented in other CME formats. One non-user thought the topics presented to date were appropriate. In response to interview questions about improving the Service or that might lead to more use of it, 10 physicians (2 non-users and 8 users) commented on the importance of relevant topics.

### Evidence-based approach

In interviews, all users were supportive of the evidence-based approach adopted in academic detailing. One user from a small community stated that as a group the community physicians had decided to adopt an evidence-based approach to practice and had taken up academic detailing based on that decision. We asked physicians if the evidence-based messages in academic detailing had affected their evaluation of information from other sources. Approximately half the users interviewed indicated that academic detailing had made them more critical of information from other CME programs, journal articles, and pharmaceutical representatives. In some cases, academic detailing had reinforced their critical approach.

"Academic detailing is making me not want to go to some of the more traditional sit down in a dark room, listen to specialist talk CME. I'm starting to expect more."

"We are reminded every time we go through academic detailing to make sure we question the level of evidence and how studies are done. That approach is very helpful."

"I can discuss things with them (pharmaceutical representatives) and if I have information from the Academic Detailing Service it helps to support my points for discussion."

Academic detailing was less likely to affect physicians' evaluation of advice from specialists because they considered specialists to be well informed of the evidence.

"Most of the specialist reports I see try to include evidence."

### Handout material

All users except 1 found the handout material useful. They appreciated the point form format of the resource booklet and the key messages found in the laminated sheet. Ten users reported they referred to the handout material for therapeutic recommendations, medication doses, and in preparation for patients who had appointments for conditions covered in the material.

"Your handouts were just so wonderful, they summarized everything so well. They're very concise and up to date."

"I have found myself referring to it on several occasions, so I'm glad I have it."

When asked for suggestions to improve the handout material, 6 users had no suggestions because they liked the existing format. Suggestions from other users were to add color and pictures, put them in format for a personal digital assistant, and include patient education material.

Users also indicated they had made practice changes based on information from academic detailers. Examples were ordering spirometry to diagnose chronic obstructive pulmonary disease, more diligent screening for osteoporosis, prescribing alendronate instead of etidronate for osteoporosis, and not prescribing rofecoxib because of concern over adverse cardiovascular effects.

Most information about ways to make the Academic Detailing Service better meet learning needs came from the user group. The most frequent suggestion was to provide the service more often (3 interviews, 8 questionnaire respondents) followed by having group sessions (2 interviews, 6 questionnaire respondents). Most comments expressed satisfaction with the Service. When asked for their general impressions in interviews, all but 1 user made favourable comments about the Service. When asked for suggestions for improvements, 3 interview informants and 49 questionnaire respondents, all from the user group, said they were satisfied with the service as it is.

## Discussion

This study identified several factors that encourage and discourage FPs from using academic detailing. We found few other studies that provide similar information. Janssens conducted interviews with physicians who did and did not use academic detailing[5] and Van Eijk listed some reasons for non-participation in an academic detailing project[11]. Soumerai and Avorn list 8 techniques of effective academic detailing based on information from pharmaceutical representatives and their own experiences[12].

In our study, spending office time doing CME was the factor that most deterred physicians, a consistent finding from interviews and the questionnaire. In interviews with non-users, physicians who saw the value of academic detailing just did not have time. In contrast, users of academic detailing did not find time to be enough of a barrier to discourage participation. Van Eijk and Janssens both identified lack of time as a barrier to participation in academic detailing[5,11]. This finding was unexpected since we thought physicians would view office-based CME as being convenient and efficient.

**Figure 3:**
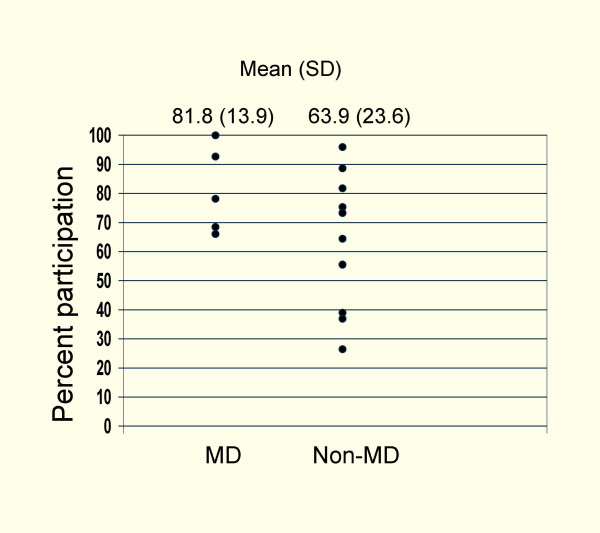
Percent participation in academic detailing interventions in which detailers were physicians or non-physicians.

Another major barrier for non-users was having non-physicians deliver the educational material. This finding was more pronounced in the questionnaire responses than in the interviews and was not identified in Janssens' study. However, Van Eijk mentioned that some physicians did not participate because her project was initiated by the school of pharmacy rather than the Faculty of Medicine, even though the detailer was a physician. We reviewed 28 studies published since 1997 to try to determine if participation in academic detailing is greater if the detailer is a physician. Including our own study, 9 studies had physicians as detailers, 16 had non-physicians, 2 had both, and 1 did not specify. Figure 3 shows participation rates for those studies that gave this information (MD detailer mean participation = 81.8% SD 13.9, non-MD detailer participation = 63.9% SD 23.6). There appears to be a trend toward higher participation in those studies in which the detailer was a physician; however a Mann-Whitney U test found no statistically significant difference, perhaps because of the small number of studies or because we did not adjust for potential confounders such as the relevance of the topic presented. It may be preferable to have a physician as detailer since this might entice non-users to participate and is unlikely to deter regular users. However, it would increase cost. This subject requires more study.

The relevance of the topics detailed was the main encouraging factor for users on the questionnaire and was mentioned by several physicians in interviews as a factor that might lead to use of academic detailing. At the time of our study, the topics we had presented were updates on influenza and pneumococcal vaccine, hormone replacement therapy, osteoarthritis, osteoporosis, and chronic obstructive pulmonary disease. These are quite conventional topics; however, we were able to bring something new to them all. For instance, in the session on chronic obstructive pulmonary disease we pointed out that some recommendations in the Canadian guidelines[13] are based on studies that show statistical significance but not clinical significance based on the scales used for outcomes. There did not appear to be a consensus as to whether we should present topics that are commonly presented in CME programs or that are somewhat unusual.

The handout material we leave with physicians was also mentioned as an encouraging factor. Our handout material is somewhat atypical for an academic detailing program. Soumarai recommends brief, graphic print materials[12]. We do produce such material in our single page laminate, however we also produce an extensive review of the evidence with several summary pages. Information from this study indicates that physicians find such information helpful and refer back to it. In a previous evaluation survey (unpublished data), 75% of 106 respondents found the booklet somewhat or very useful while 65% found the laminate useful. These findings challenge the accepted approach to producing brief academic detailing material.

One of the most interesting findings from our study was the value that FPs place on the evidence-based approach of academic detailing. Previous studies indicate that FPs use evidence-based summaries and guidelines but do not believe that learning the skills of evidence-based medicine is the best way to implement it in practice[14]. Our approach is to do the first three As of evidence-based medicine (Ask the appropriate question, Access the appropriate information, and Appraise the information)[15]. We then present our findings to physicians by providing them with information in absolute and relative terms as recommended at a recent meeting of the U.S. National Institutes of Health[16]. We explain these terms to the physicians and let them decide about the final two As (Apply the information as they think appropriate, and Assess the results.) Our goal is similar to that of Habraken et al whose underlying aim was to stimulate a critical attitude in physicians by discussing the results of studies[4]. Habraken et al speculated that physicians could apply this critical attitude to other sources of information such as pharmaceutical representatives. Our results suggest that this is the case and extends to other sources such as CME programs and journal articles. However, they are not necessarily more likely to critically appraise advice from specialists. Since specialists provide most CME there is some inconsistency in this finding and it too requires more study.

In our study, users of academic detailing value it more highly than other CME formats. This is consistent with evaluations we receive on detailing visits which consistently average at least 4.5 on a five-point Likert scale. In addition, users are much more likely than non-users to participate in the program in the future.

The main limitation of this study is the low response rate for the non-user group in both the interviews and the questionnaire. Only 15% of non-users returned the questionnaire, so our findings for this group may not be representative of the overall population of physicians who choose not to use the Service. Also, non-users who were interviewed came from the same group of physicians who returned the questionnaire further limiting the generalizability of data from this group. Unfortunately we do not know how to encourage non-users to participate in this type of research. Additional research is needed to explore perceptions of non-users in greater depth to determine if these findings can be generalized.

Another limitation of this study is that it deals with physicians' perceptions of the Service and their reasons for using or not using it. With the cross sectional design and the measures that we used, it is not possible to discern if their perceptions reflect reality. More objective measures and experimental designs could be used to determine this.

Finally, the interviewer was associated with the department that offers the Service. Although she was not involved with the Service directly, it is possible that her connection with the university department may have influenced physicians' responses during the interviews.

As a result of this study we have made few changes to our Service. We have given large group didactic presentations to try to reach physicians who do not have time to see academic detailers at their offices. Also, we are mailing key points of our academic detailing messages to non-users and giving them an opportunity to receive a list of their patients from the provincial drug insurance plan for whom the points may apply. They can use the list to see if their patients are on appropriate therapy. We are now conducting a study to determine the efficacy of this format. We have maintained our comprehensive evidence-based approach since physicians value it highly and now provide them with a brief peer-reviewed explanation of the differences between relative and absolute terms[17].

## Conclusion

Physicians who use academic detailing rate its educational value highly. Selecting relevant topics appears to be the most important factor in encouraging use of academic detailing but we did not find consensus on what type of topics physicians consider valuable. Other factors encouraging participation are adopting an evidence-based approach and providing useful handout material. In our study, physicians found comprehensive as well as concise handout material useful, a finding that challenges the tenet that handout material should be brief.

The 3 factors that most discouraged the use of academic detailing were spending office time doing CME, scheduling time to see the academic detailer, and having CME provided by a non-physician. Because we found indications that an evidence-based approach can lead to more critical thinking and practice change, it is important to consider other ways to reach non-users who find it inconvenient to spend office time doing CME. The relative merits of having physicians or non-physicians provide academic detailing require further study.

## Competing interests

Michael Allen is Director and Isobel Fleming is Senior Detailer of the Dalhousie Academic Detailing Service.

## Authors' contributions

MA conceived the project, wrote the grant proposal, helped with experimental design and data analysis, and wrote the draft of the paper. SF helped with experimental design and data analysis, wrote parts of the draft of the paper and edited the paper. NO helped with data analysis, wrote parts of the draft of the paper and edited other parts. IF helped with experimental design and edited the paper. All authors have read and approved the final manuscript.

## Pre-publication history

The pre-publication history for this paper can be accessed here:



## Supplementary Material

Additional file 1 Interview questions. These are the guiding questions for telephone interviews with physicians to determine their attitudes toward academic detailing. Click here for file
