# Supplementary material for: Family physicians' perceptions of academic detailing: a quantitative and qualitative study
Source: BMC Med Educ. 2007 Oct 12;7:36. doi: 10.1186/1472-6920-7-36 (PMC2099423; doi:10.1186/1472-6920-7-36)
Supplement: Additional file 1 — Interview questions. These are the guiding questions for telephone interviews with physicians to determine their attitudes toward academic detailing. [file 1472-6920-7-36-S1.doc]

### Family Physicians’ Perceptions of Academic Detailing:

### A Quantitative and Qualitative Study

# Interview Questions

1. What forms of CME do you find most useful?
2. Could you tell me what you know about the Dalhousie Academic Detailing Service?
   - How many times, if any, have you seen an Academic Detailer? For which topics (Influenza/flu vaccine, Osteoarthritis, HRT, Osteoporosis, COPD)?
3. What are your opinions of the Dalhousie Academic Detailing Service? (For those with experience: What did you like? What did you not like?)

Prompts:

- Do you have thoughts on the evidence-based approach used in Academic Detailing?
  - has it affected the way you evaluate articles you read?
  - has it affected the way you evaluate the content of CME programs?
  - has it affected the way you evaluate advice from specialists?
  - has it affected the way you evaluate messages from pharmaceutical reps?
  - has it affected the way you practice?
- What types of things are likely to encourage you to change your practice?
- Spending office time to see the Academic Detailer
- Scheduling a time to see the Academic Detailer
- You may be aware that our academic detailers are either a pharmacist or a nurse. How do you feel about having medical education provided by a non-MD?

1. **USED NEVER**: Could you tell me why you have never used the Dalhousie Academic Detailing Service?

**USED ONCE**: Could you tell me why you have used the Dalhousie Academic Detailing Service only once?

**USED >ONCE**: Could you tell me why you have used the Dalhousie Academic Detailing Service several times?

1. What might encourage you to use the Dalhousie Academic Detailing Service (again)?
2. Do you have any suggestions for the Dalhousie Academic Detailing Service?

- Some complex topics include a lot of detailed information. What do you think of having 2 consecutive visits, 2 or 3 months apart, on the same topic (e.g., diabetes) but having each visit concentrate on different aspects (e.g., evidence for effectiveness of home glucose monitoring; evidence for new oral agents)?

1. **USED ONCE & >ONCE**: Did you receive a handout when visited by the academic detailer?

- Were the handout materials helpful during the Academic Detailing visit?
- Do you still refer to the materials? If so, please discuss how often and under what circumstances.
- Do you have any suggestions for improving the content (e.g., depth and breadth of information) and format (layout) of the materials?
- Are you aware of the summary statements at the beginning of the handout. Do you use them for key messages?

**USED NEVER**: What type of handout would you find useful?

- What are some examples of handouts from other sources that you use on a regular basis?

1. Do you have any other comments?
